# Antifungal activity of redox-active benzaldehydes that target cellular antioxidation

**DOI:** 10.1186/1476-0711-10-23

**Published:** 2011-05-31

**Authors:** Jong H Kim, Kathleen L Chan, Noreen Mahoney, Bruce C Campbell

**Affiliations:** 1 Plant Mycotoxin Research Unit, Western Regional Research Center, USDA-ARS, 800 Buchanan St., Albany, CA 94710, USA

## Abstract

**Background:**

Disruption of cellular antioxidation systems should be an effective method for control of fungal pathogens. Such disruption can be achieved with redox-active compounds. Natural phenolic compounds can serve as potent redox cyclers that inhibit microbial growth through destabilization of cellular redox homeostasis and/or antioxidation systems. The aim of this study was to identify benzaldehydes that disrupt the fungal antioxidation system. These compounds could then function as chemosensitizing agents in concert with conventional drugs or fungicides to improve antifungal efficacy.

**Methods:**

Benzaldehydes were tested as natural antifungal agents against strains of *Aspergillus fumigatus*, *A. flavus*, *A. terreus *and *Penicillium expansum*, fungi that are causative agents of human invasive aspergillosis and/or are mycotoxigenic. The yeast *Saccharomyces cerevisiae *was also used as a model system for identifying gene targets of benzaldehydes. The efficacy of screened compounds as effective chemosensitizers or as antifungal agents in formulations was tested with methods outlined by the Clinical Laboratory Standards Institute (CLSI).

**Results:**

Several benzaldehydes are identified having potent antifungal activity. Structure-activity analysis reveals that antifungal activity increases by the presence of an *ortho*-hydroxyl group in the aromatic ring. Use of deletion mutants in the oxidative stress-response pathway of *S. cerevisiae *(*sod1*Δ, *sod2*Δ, *glr1*Δ) and two mitogen-activated protein kinase (MAPK) mutants of *A. fumigatus *(*sakA*Δ, *mpkC*Δ), indicates antifungal activity of the benzaldehydes is through disruption of cellular antioxidation. Certain benzaldehydes, in combination with phenylpyrroles, overcome tolerance of *A. fumigatus *MAPK mutants to this agent and/or increase sensitivity of fungal pathogens to mitochondrial respiration inhibitory agents. Synergistic chemosensitization greatly lowers minimum inhibitory (MIC) or fungicidal (MFC) concentrations. Effective inhibition of fungal growth can also be achieved using combinations of these benzaldehydes.

**Conclusions:**

Natural benzaldehydes targeting cellular antioxidation components of fungi, such as superoxide dismutases, glutathione reductase, *etc*., effectively inhibit fungal growth. They possess antifungal or chemosensitizing capacity to enhance efficacy of conventional antifungal agents. Chemosensitization can reduce costs, abate resistance, and alleviate negative side effects associated with current antifungal treatments.

## Background

A number of different cellular targets of conventional antifungal drugs have already been identified. Examples include mitochondrial respiration, cell wall/membrane integrity, cell division, signal transduction, and macromolecular synthesis, *etc*. [1]. However, conventional antifungal drugs (including fungicides) also cause serious mammalian cytotoxicity, partly through the intracellular production of reactive oxygen species (ROS) [2]. Emerging resistance to currently available antifungal drugs and a deficiency in discovery of new ones engender urgency for development of new antifungal agents and/or alternative therapies for control of fungal pathogens [3-7].

Natural compounds that do not have any significant medical or environmental impact are a potential source of antimycotic agents, either in their nascent form or as template structures for more effective derivatives [8,9]. Prior studies showed that analogs of benzoic or cinnamic acids, common phenolics found in edible plants, inhibit biosynthesis of mycotoxins and growth of various fungi, both filamentous and yeasts [10-12]. Noteworthy is that these phenolics can be potent redox cyclers that inhibit microbial growth through disruption of cellular redox homeostasis and/or antioxidation systems [13,14].

From a clinical perspective, the functions of antioxidation systems [*e. g*., mitogen-activated protein kinases (MAPKs)], two-component histidine kinase and antioxidation enzymes [*e.g*., superoxide dismutases (SODs), catalase, *etc*.], have been implicated as factors important to the virulence of fungal pathogens [15,16]. In *Aspergillus fumigatus*, Cu,Zn-SOD detoxifies ROS produced by host defense systems [17]. Fungi require well defined regulation of expression of antioxidation systems, not only for protection from host defense responses, but also for maintaining redox homeostasis needed for normal fungal growth [18,19]. Because of this pivotal role, destabilization of antioxidation systems can be an effective way to control fungal pathogens. Such destabilization may be possible with redox-active compounds.

Inhibitors of the mitochondrial respiratory chain (MRC), such as antimycin A or mucidin, disrupt cellular energy production in fungi [20,21], decreasing cell viability. Coinciding with this disruption is an abnormal release of electrons from the chain. This surfeit of electrons further stresses cellular components through oxidative damage resulting in apoptosis or necrosis [21,22]. As indicated above, the cellular antioxidation system [*e.g*., cytosolic superoxide dismutase (Cu,Zn-SOD), mitochondrial superoxide dismutase (Mn-SOD), glutathione reductase, *etc*.] plays an important defensive role in protecting fungal cells from such oxidative species [23,24].

Other studies have also shown that antimicrobial activity of a variety of drugs can be linked to cellular oxidative stress. Examples include ciprofloxacin, a fluoroquinolone antibiotic inhibiting DNA topoisomerases ([25] and references therein). After treatment of ciprofloxacin, the level of ROS was increased in bacterial pathogens. However, application of antioxidants, such as reduced glutathione (GSH) or ascorbic acid, reversed the toxicity of fluoroquinolones. In addition, transfection of SOD genes into bacteria also resulted in greater survival of cells exposed to these drugs [25], indicating ROS (*i.e*., superoxides, peroxides, *etc*.) are involved in antimicrobial activity of ciprofloxacin. Amphotericin B (AMB), a polyene antifungal drug, is another example. Although AMB is known as a fungicidal drug, studies have shown that addition of antioxidants, such as GSH, cysteine, *etc*., could revive endospores of *Coccidioides immitis *treated with AMB ([26] and references therein). Other data also indicate involvement of cellular oxidative stress in the antifungal action of AMB [27,28].

Co-application of certain types of compounds can enhance effectiveness of conventional antimicrobial agents through a process termed chemosensitization. In this case, a chemosensitizing agent functions by debilitating the ability of a pathogen to completely activate a defense response to an antimicrobial agent [29,30]. A chemosensitizing agent does not necessarily require a great degree of antimicrobial potency, itself, to be effective.

The chief value of chemosensitization, especially by safe natural compounds, is lowering of dosage levels of commercial drugs required for control of pathogens; thus, lowering costs and risks of negative side effects. Redox-active natural compounds that destabilize the fungal antioxidation system could act as potent chemosensitizing agents when co-applied with oxidative stress drugs, such as MRC inhibitors, for control of fungal pathogens. Thus, chemosensitization could make the use of toxic antifungal drugs or fungicides more attractive as a chemotherapeutic strategy, and to overcome development of pathogen resistance to conventional antimicrobial agents.

Filamentous fungi in the genus *Aspergillus *are notable etiological agents of a highly debilitating human disease, invasive aspergillosis [31]. Among these are *A. fumigatus*, *A. terreus *and *A. flavus*, ubiquitous opportunistic pathogens. *A. flavus *also produces hepatocarcinogenic aflatoxins that are a major food safety issue in that they can contaminate a variety of edible crops and their by-products [32]. Likewise, another mycotoxin, patulin [4-hydroxy-4*H*-furo (3,2C) pyran-2(6*H*)-one], that can contaminate fruits, causes serious acute/chronic cellular or target-organ toxicity in mammals by disrupting cellular [33,34] and enzymatic [35,36] processes. Patulin is most commonly produced by fungi in the genera *Aspergillus *and *Penicillium *[37,38]. Among these, *P. expansum *is of highest food safety concern with regard to patulin-production.

The yeast *Saccharomyces cerevisiae *is a useful model system for identifying antifungal agents and their gene targets in view that: (1) the genome of *S. cerevisiae *has been sequenced and well annotated ([39], accessed March 1, 2011); (2) *S. cerevisiae *gene deletion mutants have proven to be very useful for identifying the mechanism/target genes of antimicrobial agents [40]; and (3) many genes in *S. cerevisiae *are orthologs of genes of fungal pathogens [41]. For example, we recently confirmed structural homology of signal transduction and antioxidation genes between *S. cerevisiae *and the filamentous fungus *A. flavus *[42].

In this study, we identify safe natural phenolics, which can specifically disrupt the fungal antioxidation system. As potent redox cyclers, phenolic compounds can effectively debilitate the cellular redox homeostasis/antioxidation system in fungi, resulting in the suppression of fungal growth. We describe a bioassay, using *S. cerevisiae *as a model, which provides a framework for examining structure-activity relationships of screened compounds and for identifying promising molecular targets. In this study, we focus on effectiveness of structural analogs of benzaldehyde to identify: (1) the most effective antifungal target in the mitochondrial respiratory chain (MRC); (2) the most effective analogs as antifungal agents; and (3) the fungal antioxidation system as the target of the benzaldehydes. In addition, we examine if benzaldehydes can serve as chemosensitizing agents or as antifungal agents, in combination amongst themselves, at the micromolar level.

## Methods

### Microorganisms

*Aspergillus fumigatus *AF293 (wild type), and *A. fumigatus *MAPK deletion mutants *sakA*Δ and *mpkC*Δ [43,44] were grown at 35°C on potato dextrose agar (PDA). *A. terreus *UAB673, UAB680 and UAB698, clinical strains from aspergillosis patients, were obtained from Centers for Disease Control and Prevention, Atlanta, GA, and were grown at 35°C on PDA. Also, *A. flavus *NRRL3357 and *Penicillium expansum *NRRL974, obtained from the National Center for Agricultural Utilization and Research, USDA-ARS, Peoria, IL, were grown at 28°C on PDA. Temperatures used were the optimum temperatures for each strain. *Saccharomyces cerevisiae *wild type BY4741 (*Mat *a *his3*Δ*1 leu2*Δ*0 met15*Δ*0 ura3*Δ*0*) and selected single gene deletion mutants, *i.e*., cytosolic superoxide dismutase (Cu,Zn-SOD) mutant (*sod1*Δ), mitochondrial superoxide dismutase (Mn-SOD) mutant (*sod2*Δ) and glutathione reductase mutant (*glr1*Δ), were obtained from Invitrogen (Carlsbad, CA) and Open Biosystems (Huntsville, AL; [39], accessed March 1, 2011). Yeast strains were cultured on SG (Yeast nitrogen base without amino acids 0.67%, glucose 2% with appropriate supplements: uracil 0.02 mg/ml, amino acids 0.03 mg/ml) agar at 30°C.

### Chemicals

Benzaldehyde (basal compound) and its structural analogs (twenty-one new benzaldehyde derivatives), *i.e*., cinnamaldehyde, 2-hydroxy-3-methoxybenzaldehyde (*o*-vanillin), 2-hydroxy-5-methoxybenzaldehyde, 2-methylbenzaldehyde (*o*-tolualdehyde), 3-methylbenzaldehyde (*m*-tolualdehyde), 4-methylbenzaldehyde (*p*-tolualdehyde), 2-methoxybenzaldehyde (*o*-anisaldehyde), 3-methoxybenzaldehyde (*m*-anisaldehyde), 4-methoxybenzaldehyde (*p*-anisaldehyde), 2,3-dimethoxybenzaldehyde, 2,4-dimethoxybenzaldehyde, 2,5-dimethoxybenzaldehyde, 3,5-dimethoxybenzaldehyde, 2,4,5-trimethoxybenzaldehyde, 4-hydroxy-2-methoxybenzaldehyde, 3,5-dimethoxy-4-hydroxybenzaldehyde (syringaldehyde), 3,5-dimethoxy-4-hydroxycinnamaldehyde, 4-methoxy-2-methylbenzaldehyde, 2-hydroxy-5-methylbenzaldehyde, 2,4-dimethylbenzaldehyde, 4-diethylamino-2-hydroxybenzaldehyde, two phenolics (cell wall/membrane integrity disruptors) [2,3-dihydroxybenzaldehyde (2,3-D), thymol], strobilurins [pyraclostrobin (PCS), kresoxim methyl (Kre-Me)] and other chemicals [antimycin A (AntA), carboxin, thenoyltrifluoroacetone (TTFA), rotenone, 3-nitropropionic acid (3-NPA), benzhydroxamic acid (BHAM), salicylhydroxamic acid (SHAM), potassium cyanide (KCN), fludioxonil, glutathione (GSH, reduced form; GSSG, oxidized form), dimethyldithiocarbamate (DDC)] were procured from Sigma Co. (St. Louis, MO, USA). Each compound was dissolved in dimethylsulfoxide (DMSO; absolute DMSO amount: < 2% in media), except glutathione, which was dissolved in water, before incorporation into culture media. In all tests, control plates (*i.e*., "No treatment") contained DMSO at levels equivalent to that of cohorts receiving antifungal agents, within the same set of experiments (See Figures).

### Antifungal bioassays

#### Plate (agar) bioassay

Measurement of sensitivities of filamentous fungi to the structural analogs of benzaldehyde was based on percent radial growth of treated compared to control fungal colonies (Test concentrations: 0, 0.5, 1.0, 1.5, 2.0, 2.5, 3.0 mM). The percent inhibition of growth was calculated using the Vincent equation [45] [% inhibition = 100 (C-T)/C; where C = diameter of fungal colony on control plate (receiving only DMSO), and T = diameter of fungal colony on the treated plate]. Minimum Inhibitory Concentration (MIC) values on agar plates were based on triplicate assays and defined as the lowest concentration of agent where no fungal growth was visible on the plate. For the above assays, fungal conidia (5 × 10^3^) were diluted in phosphate buffered saline and applied as a drop onto the center of PDA plates with or without antifungal compounds. Growth was observed for three to seven days.

Petri plate-based yeast dilution bioassays were performed on the wild type and antioxidation mutants (*sod1*Δ, *sod2*Δ, *glr1*Δ) to assess effects of screened compounds on the antioxidation system. Yeast strains were exposed to 0.1 to 1.5 mM of seven benzaldehyde analogs screened. These assays were performed in duplicate on SG agar following previously described protocols [46]

#### Microdilution (microtiter) bioassay

Levels and types of compound interactions between antifungal agents were based on Fractional Inhibitory Concentration Indices (FICI) [47], where FICI = (MIC of compound A in combination with compound B/MIC of compound A, alone) + (MIC of compound B in combination with compound A/MIC of compound B, alone). Compound interactions were defined as synergistic (FICI ≤ 0.5), additive (0.5 < FICI ≤ 1), neutral (1 < FICI ≤ 2) or antagonistic (2 < FICI).

To determine antifungal MICs in microtiter wells for use in calculating FICIs, triplicate assays (4 × 10^4 ^conidia/ml) were performed using a broth microdilution according to methods outlined by the Clinical Laboratory Standards Institute (CLSI) M38-A [48]. RPMI 1640 medium (Sigma Co.) was supplemented with 0.03% L-glutamine and buffered with 0.165 mM 3-[*N*-morpholino] propanesulfonic acid. Concentrations of test compounds used for chemosensitization assays were as described in the text.

### Formulation studies of benzaldehydes

Formulation studies included *o*-vanillin, 2-hydroxy-5-methoxybenzaldehyde, cinnamaldehyde, which showed the highest antifungal activities (Group A benzaldehydes: See below) and two additional phenolic agents, 2,3-dihydroxybenzaldehyde (2,3-D) and thymol, found in a prior study to disrupt cell wall/membrane integrity [46]. Compounds were tested either singularly or combined in formulations that included all five compounds. Antifungal efficacies of singular compounds *vs*. formulations were compared. Treatments included the compounds at 2, 4, 8, 16, 32, 64 or 128 μg/ml alone, or all together in equal amounts at these same concentrations (*e.g*., mixtures of 2 μg/ml, each, 4 μg/ml, each, *etc*., of all five compounds), to determine MICs and Minimum Fungicidal Concentrations (MFCs) of the compounds individually and as formulations. All assays were performed in triplicate in 96-well microtiter plates (48 hrs incubation for determining MICs).

The level of antifungal efficacy of a formulation was evaluated by determining ratio of MFC/MIC. To obtain MFC values, the entire volume of each well (200 μl per well) from microtiter plates (at 48 hrs of incubation) was spread onto independent PDA plates, and cultured for an additional 48 hrs at temperatures respective for each fungus (See above). MFC was defined as the lowest concentration of agent where > 99.9% fungal death occurred, as determined by cell growth on the agar plate. If MFC_FORMULATION_/MIC_FORMULATION _was ≤ 4, the formulation was defined as fungicidal, whereas if MFC_FORMULATION_/MIC_FORMULATION _was > 4, the formulation was defined as fungistatic [49].

Assessment of compound formulations was measured by formulation efficacy (FE), *i.e*., FE_MIC _or FE_MFC_. Respective FEs were calculated, as follows: FE_MIC_= (MIC of compound A, alone/MIC_FORMULATION_) or FE_MFC _= (MFC of compound A, alone/MFC_FORMULATION_), respectively. We defined FE as (a) no effect, if FE ≤ 1, (b) low (L), if 1 < FE < 4, or (c) high (H), if FE ≥ 4 (See also table 4).

## Results

### Complexes II and III as the most effective antifungal targets in the mitochondrial respiratory chain (MRC)

**Figure 1:**
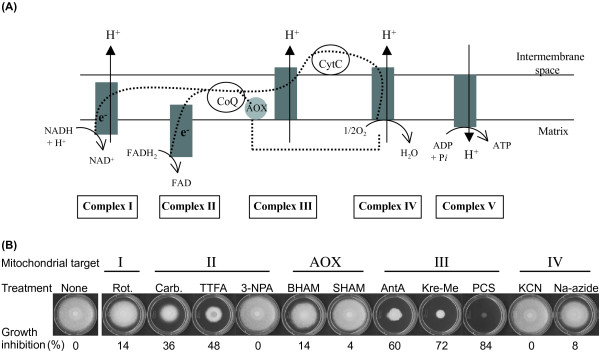
**Targeting the mitochondrial respiratory chain**. **(A) **Schematic representation of mitochondrial respiratory chain (Adapted from [78] and [22]. CoQ, Coenzyme Q; CytC, Cytochrome C; e^-^, Electrons; Dashed lines, Electron flow. **(B) **Differential antifungal efficacy of inhibitors (0.1 mM) of mitochondrial respiration targeting complexes I to IV and alternative oxidases (AOX), tested in *Aspergillus fumigatus *AF293. Results indicated that targeting complex II or III by using carboxin, TTFA, antimycin A, kresoxim methyl or pyraclostrobin resulted in higher inhibition of fungal growth than targeting other complexes (%: Growth inhibition rate, SD < 5%). Rot, Rotenone; Carb, Carboxin; TTFA, Thenoyltrifluoroacetone; 3-NPA, 3-Nitropropionic acid; BHAM, Benzhydroxamic acid; SHAM, Salicylhydroxamic acid; AntA, Antimycin A; Kre-Me, Kresoxim methyl; PCS, Pyraclostrobin; KCN, Potassium cyanide; Na-azide, Sodium azide. I to IV, complexes I to IV of mitochondrial respiratory chain; AOX, Alternative oxidases.

Since various antifungal agents inhibit different components of the MRC at varying levels, we initially attempted to identify the most effective antifungal target(s) within this chain. We tested eleven conventional antifungal agents, which disrupt the functions of complexes I, II, III, IV or alternative oxidases (AOX) in the MRC, using *A. fumigatus *AF293 as a representative fungal pathogen (Figure 1A).

Targeting MRC complex II of *A. fumigatus *AF293 with thenoyltrifluoroacetone (TTFA) or carboxin, or complex III with antimycin A (AntA) or strobilurins [*i.e*., kresoxim methyl (Kre-Me)/pyraclostrobin (PCS)], resulted in greater inhibition of fungal growth (Vincent equation: 36% - 84%) compared to targeting other MRC complexes (Vincent eq.: 0% - 14%) (Figure 1B). Results also indicated that complex III inhibitors possessed higher antifungal activity (Vincent eq.: 60% - 84%) than complex II inhibitors (Vincent eq.: 36% - 48%). In our test, the complex II inhibitor 3-nitropropionic acid (3-NPA) had no discernable growth inhibitory effect on *A. fumigatus*. Neither did the complex I inhibitor, rotenone (Rot.), AOX inhibitors, benz- or salicyl-hydroxamic acids (BHAM, SHAM), nor complex IV inhibitors potassium cyanide (KCN) and sodium azide (Na-azide) (Figure 1B). DMSO is a good solvent for polar and non-polar compounds, is miscible in varying organic substances and is widely used to enhance absorption for drug-delivery [50]. We considered that the individual MRC inhibitors tested were absorbed at equivalent levels by fungal cells. However, future studies should specifically examine if these inhibitors reach targets within cells at the same rates and levels.

To confirm if inhibitors of complexes II and III can function synergistically to disrupt fungal mitochondrial respiration, TTFA/carboxin (complex II inhibitors) and AntA/Kre-Me/PCS (complex III inhibitors) were co-applied against six strains of filamentous fungal pathogens, *i.e*., *A. fumigatus*, the three *A. terreus *strains, *A. flavus*, and *P. expansum *(Table 1). Co-treatment of complex II and III inhibitors greatly increased their antifungal activities in comparison to each compound, alone. Most compound FICI-based interactions were either additive or synergistic, depending on types of drug combinations and/or strains tested. For example, interactions between carboxin with any of the complex III inhibitors were synergistic in all fungi tested, except with AntA in *A. flavus*, where it was additive. Interactions between TTFA and the complex III inhibitors were, for the most part, synergistic as well. The exceptions were additive interactions with AntA in *A. terreus *UAB673 and *A. fumigatus *and a neutral interaction in *A. flavus*. Collectively, results indicated that targeting complexes II and III of the MRC, simultaneously, could prove to be an effective antifungal strategy, in that their inhibitors mainly act synergistically.

**Table 1:** Antifungal interactions (FICI) between complex II and III inhibitors tested against filamentous fungi in microtiter plates^1^.

Combinations A	MIC alone	MIC combined	FICI	MIC alone	MIC combined	FICI	MIC alone	MIC combined	FICI
**Strains**	***A. terreus *UAB698**			***A. terreus *UAB680**			***A. terreus *UAB673**		
Kre-Me Carboxin	> 1.6^2^ > 128^3^	0.2 - 0.4 8 - 16	0.19 S	> 1.6 > 128	0.2 - 0.4 4 - 8	0.16 S	> 1.6 > 128	0.2 - 0.4 8 - 16	0.19 S
Pyraclostrobin Carboxin	> 1.6 > 128	0.0 - 0.05 4 - 8	0.05 S	> 1.6 > 128	0.0 - 0.05 4 - 8	0.05 S	> 1.6 > 128	0.0 - 0.05 4 - 8	0.05 S
AntimycinA Carboxin	> 1.6 > 128	0.4 - 0.8 8 - 16	0.31 S	> 1.6 > 128	0.4 - 0.8 16 - 32	0.38 S	> 1.6 > 128	0.4 - 0.8 16 - 32	0.38 S

**Strains**	***A. flavus *NRRL3357**			***A. fumigatus *AF293**			***P. expansum *NRRL974**		
Kre-Me Carboxin	0.4 - 0.8 > 128	0.0 - 0.05 0.5 - 1	0.07 S	> 1.6 > 128	0.0 - 0.05 2 - 4	0.03 S	0.4 - 0.8 32 - 64	0.0 - 0.05 0.5 - 1	0.08 S
Pyraclostrobin Carboxin	0.4 - 0.8 > 128	0.0 - 0.05 0.125 - 0.25	0.06 S	0.8 - 1.6 32 - 64	0.0 - 0.05 1 - 2	0.06 S	0.4 - 0.8 2 - 4	0.0 - 0.05 0.25 - 0.5	0.19 S
AntimycinA Carboxin	0.4 - 0.8 > 128	0.2 - 0.4 4 - 8	0.53 A	0.8 - 1.6 > 128	0.2 - 0.4 4 - 8	0.28 S	0.4 - 0.8 > 128	0.05 - 0.1 2 - 4	0.14 S

**Combinations B**	**MIC alone**	**MIC Combined**	**FICI**	**MIC alone**	**MIC combined**	**FICI**	**MIC alone**	**MIC combined**	**FICI**
**Strains**	***A. terreus *UAB698**			***A. terreus *UAB680**			***A. terreus *UAB673**		
Kre-Me TTFA	> 1.6^2^ > 128^3^	0.2 - 0.4 4 - 8	0.16 S	> 1.6 > 128	0.2 - 0.4 16 - 32	0.25 S	> 1.6 > 128	0.2 - 0.4 16 - 32	0.25 S
Pyraclostrobin TTFA	> 1.6 > 128	0.1 - 0.2 4 - 8	0.09 S	> 1.6 > 128	0.2 - 0.4 4 - 8	0.16 S	> 1.6 > 128	0.2 - 0.4 4 - 8	0.16 S
AntimycinA TTFA	> 1.6 > 128	0.4 - 0.8 32 - 64	0.50 S	> 1.6 > 128	0.4 - 0.8 32 - 64	0.50 S	> 1.6 > 128	0.8 - 1.6 32 - 64	0.75 A

**Strains** **Compounds**	***A. flavus *NRRL3357**			***A. fumigatus *AF293**			***P. expansum *NRRL974**		
Kre-Me TTFA	> 1.6 > 128	0.4 - 0.8 16 - 32	0.38 S	> 1.6 > 128	0.2 - 0.4 8 - 16	0.19 S	> 1.6 16 - 32	0.4 - 0.8 4 - 8	0.50 S
Pyraclostrobin TTFA	> 1.6 > 128	0.4 - 0.8 8 - 16	0.31 S	> 1.6 > 128	0.1 - 0.2 4 - 8	0.09 S	> 1.6 4 - 8	0.05 - 0.1 1 - 2	0.28 S
AntimycinA TTFA	> 1.6 > 128	> 1.6 > 128	1.00 N	> 1.6 > 128	0.8 - 1.6 32 - 64	0.75 A	> 1.6 > 128	0.00 - 0.05 8 - 16	0.08 S

^1 ^Compound interactions were determined as Fractional Inhibitory Concentration Indices (FICI), described by Isenberg ([47]; See also Methods). For calculation purposes, the higher concentration in each column was used. A, additive; N, neutral; S, synergistic. Complex II inhibitors (μg/ml)- Carboxin, Thenoyltrifluoroacetone (TTFA); Complex III inhibitors (mM)- Kresoxim methyl (Kre-Me), Pyraclostrobin, Antimycin A. Drug combinations for calculating FIC indices were: (Combinations A) Kre-Me + carboxin, pyraclostrobin + carboxin, and antimycin A + carboxin, and (Combinations B) Kre-Me + TTFA, pyraclostrobin + TTFA, and antimycin A + TTFA.

^2 ^Since antifungal test was performed up to 1.6 mM of strobilurins or antimycin A (See Methods), 3.2 mM (doubling of 1.6 mM) was used for calculation purposes.

^3 ^Since antifungal test was performed up to 128 μg/ml of carboxin or TTFA (See Methods), 256 μg/ml (doubling of 128 μg/ml) was used for calculation purposes.

### Identification of benzaldehyde analogs possessing potent antifungal activities: structure-activity relationships

**Figure 2:**
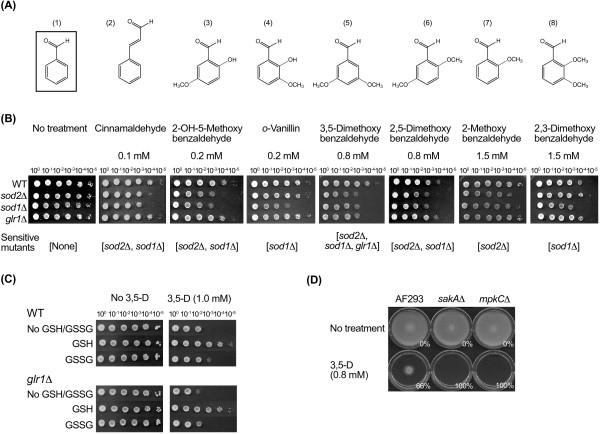
**Structure-activity relationships of the benzaldehydes in targeting the oxidative-stress response system of fungi**. **(A) **Benzaldehyde and its structural analogs used in this study. (1) Benzaldehyde (parent compound), (2) *trans*-Cinnamaldehyde, (3) 2-Hydroxy-5-methoxybenzaldehyde, (4) 2-Hydroxy-3-methoxybenzaldehyde (*o*-Vanillin), (5) 3,5-Dimethoxybenzaldehyde, (6) 2,5-Dimethoxybenzaldehyde, (7) 2-Methoxybenzaldehyde, (8) 2,3-Dimethoxybenzaldehyde. **(B) **Responses of *Saccharomyces cerevisiae *wild type and antioxidation mutant strains to the treatment of benzaldehyde analogs. Sensitive mutants in each treatment were designated under the yeast dilution bioassay (10^0 ^to 10^5 ^indicates the dilution rate for spotting onto agar plate). **(C) **Recovery of the growth of yeast cells treated with 3,5-dimethoxybenzaldehyde (3,5-D) by reduced form of glutathione (GSH; 0.1 mM), but not by oxidized form of glutathione (GSSG; 0.1 mM). **(D) **Sensitive responses of *A. fumigatus *MAPK mutants (*sakA*Δ and *mpkC*Δ) to 3,5-dimethoxybenzaldehyde (3,5-D)(%: Growth inhibition rate, SD < 5%).

Next, we examined antifungal efficacy of 21 analogs of benzaldehyde against the six different strains/species of filamentous fungi, in *in vitro *agar plate bioassays. Seven analogs (Figure 2A) were found that had higher antifungal activity (*i.e*., MIC ≤ 3.0 mM cutoff) than other compounds. The screened compounds were categorized into four groups, based on level of antifungal activity, as follows (MICs based on average values obtained from all six filamentous fungi): Group A (0.5 < MIC ≤ 1.0 mM)- cinnamaldehyde, 2-hydroxy-3-methoxybenzaldehyde (*o*-vanillin) and 2-hydroxy-5-methoxybenzaldehyde; Group B (1.0 < MIC ≤ 2.0 mM)- 2,5-dimethoxybenzaldehyde and 3,5-dimethoxybenzaldehyde; Group C (2.0 < MIC ≤ 3.0 mM)- 2-methoxybenzaldehyde (*o*-anisaldehyde) and 2,3-dimethoxybenzaldehyde; and Group D (MIC > 3.0 mM)- the remaining 14 benzaldehyde analogs.

Structure-activity relationships were also found among the screened compounds. Firstly, the *ortho*-hydroxyl (2-OH) group on the aromatic ring, in general, results in higher antifungal activity compared with an *ortho*-methoxy (2-OMe) group (Table 2). For example, 2-hydroxy-3-methoxybenzaldehyde (*o*-vanillin; MIC 0.67 mM) has > 3-fold antifungal activity (*viz*., lower MIC) than 2,3-dimethoxybenzaldehyde (MIC 2.5 mM). Likewise, 2-hydroxy-5-methoxybenzaldehyde (MIC 0.58 mM) has again, almost three-fold greater antifungal activity than 2,5-dimethoxybenzaldehyde (MIC 1.5 mM). A similar comparative trend can be seen with the MICs of 2-hydroxy-3-methoxybenzaldehyde or 2-hydroxy-5-methoxybenzaldehyde against that of 2-methoxybenzaldehyde (MIC 2.42 mM) (Table 2). [Note: Since 2-hydroxybenzaldehyde (Salicylaldehyde) is a volatile, it was not included in this study but does have antifungal and chemosensitizing activity [51]].

**Table 2:** Antifungal activities (MIC mM) of benzaldehyde derivatives tested on agar against filamentous fungi.

Compound	*A. fumigatus* AF293	*A. terreus* UAB673	*A. terreus* UAB680	*A. terreus* UAB698	*A. flavus* NRRL3357	*P. expansum* NRRL974	Mean MIC
Cinnamaldehyde	1.0	0.5	0.5	0.5	0.5	0.5	0.58 ± 0.20^1^
2-Hydroxy-5-methoxy- benzaldehyde	0.5	0.5	0.5	0.5	0.5	1.0	0.58 ± 0.20^1^
2-Hydroxy-3-methoxy- benzaldehyde (*o*-Vanillin)	0.5	0.5	0.5	0.5	1.0	1.0	0.67 ± 0.26^1^
3,5-Dimethoxybenzaldehyde	1.0	1.0	1.0	1.0	1.5	1.5	1.17 ± 0.26^1^
2,5-Dimethoxybenzaldehyde	1.5	1.5	1.5	1.0	1.5	2.0	1.50 ± 0.32^1^
2-Methoxybenzaldehyde (*o*-Anisaldehyde)	2.0	2.5	2.5	2.5	2.5	2.5	2.42 ± 0.20^1^
2,3-Dimethoxybenzaldehyde	2.5	2.5	2.0	2.0	3.0	3.0	2.50 ± 0.45^1^
Benzaldehyde (Basal structure)	> 35.0	> 35.0	> 35.0	> 35.0	> 35.0	> 35.0	> 35.0

^1 ^*P *< 0.0005 (Student's *t *test for paired data, *i.e*., *vs*. mean MIC of benzaldehyde).

Antithetically, a methyl group, in general, reduced antifungal activities of benzaldehyde analogs (All methyl-containing compounds belong to the least active, Group D compounds). For example, 2-hydroxy-5-methylbenzaldehyde, generated by simple deoxygenation of the methoxy group of 2-hydroxy-5-methoxybenzaldehyde, showed a much higher MIC (> 3.0 mM) than the latter (MIC 0.58 mM). Likewise, the MIC of 2-methylbenzaldehyde exceeds 3.0 mM, while that of 2-methoxybenzaldehyde was 2.42 mM.

Of note is that 2,3- and 2,5-dimethoxybenzaldehyde (MICs: 2.50 and 1.50 mM, respectively) have higher antifungal activity than 2,4-dimethoxybenzaldehyde (MIC: > 3.0 mM). This result is similar to the characteristics of quinone derivatives, where functions of enzymes/proteins are inhibited mostly by those derivatives having an *ortho*- or *para*-quinonoid structure. For example, acetaminophen, a benzoquinoid, is a known inhibitor of a macrophage migration inhibitory factor tautomerase [52]. This type of inhibitory potential may explain why a methoxy group in an *ortho*- or *para*-position also results in relatively higher antifungal activity than that in a *meta*-position.

### Effect of benzaldehydes on fungal antioxidation: *S. cerevisiae *antioxidation mutants

Yeast dilution bioassays were performed using *S. cerevisiae *wild type and antioxidation mutants, *sod1*Δ, *sod2*Δ and *glr1*Δ, against the seven most active benzaldehyde analogs. Both the *sod1*Δ and *sod2*Δ mutants showed reduced cell growth as represented by a two log_10 _less dilution of yeast cells before appearance of a colony when treated with cinnamaldehyde, 2-hydroxy-5-methoxybenzaldehyde, 2,5-dimethoxybenzaldehyde and 3,5-dimethoxybenzaldehyde, compared to the wild type (Figure 2B). Additionally, the *sod1*Δ mutant showed this same level of sensitivity to *o*-vanillin and 2,3-dimethoxybenzaldehyde, while *sod2*Δ showed this sensitivity to 2-methoxybenzaldehyde, respectively.

The *glr1*Δ mutant did not show much sensitivity to any of the compounds except for 3,5-dimethoxybenzaldehyde (Figure 2B). The sensitivity to this compound is noteworthy because it suggests it disrupts cellular glutathione (*gamma*-L-Glutamyl-L-Cysteinylglycine; a cellular antioxidant) homeostasis. The role of Glr1p (glutathione reductase) is to replenish cellular GSH (a reduced form of glutathione) by reducing GSSG (an oxidized form of glutathione) [24]. We postulated the antifungal action of 3,5-dimethoxybenzaldehyde resulted from interference with the activity of Glr1p, which we investigated further, described below.

The *S. cerevisiae *wild type and *glr1*Δ strains were provided with either GSH or GSSG in the presence of 3,5-dimethoxybenzaldehyde (1.0 mM). Supplementation with GSH (0.1 mM) almost completely recovered the growth of both wild type and *glr1*Δ strains from the toxicity of 3,5-dimethoxybenzaldehyde (Figure 2C). However, supplementation with GSSG (0.1 mM) did not result in growth-recovery. These results, the growth recovery of the wild type and *glr1*Δ strains by GSH but not by GSSG, further indicate 3,5-dimethoxybenzaldehyde disrupts cellular glutathione homeostasis by interfering with Glr1p activity in fungi.

In summary, all seven of the "active" benzaldehydes targeted the cellular antioxidation system, such as Cu,Zn-SOD, Mn-SOD. In particular, one of them, 3,5-dimethoxybenzaldehyde targeted Glr1p. It thus appears these systems in fungi play an important role in responding to and/or detoxifying these benzaldehydes.

### Effect of benzaldehydes on fungal antioxidation: *A. fumigatus *MAPK mutants

In yeasts, such as *S. cerevisiae *and *Schizosaccharomyces pombe*, the regulation of *SOD1*, *SOD2 *and *GLR1 *genes is controlled by the MAPK signaling pathway, such as Hog1p [53]. SakA and MpkC in *A. fumigatus *are orthologous proteins to Hog1p of *S. cerevisiae *[43,44]. *A. fumigatus sakA*Δ is an osmotic/oxidative stress sensitive mutant, while the *mpkC*Δ is a mutant of the polyalcohol sugar utilization system [43,44]. Prior studies indicated that SakA and MpkC MAPK pathways are differentially regulated. None of the SakA-responsive cues tested, such as oxidative stressors, resulted in a common phenotype for the *mpkC*Δ mutant [43,44]. Hence, it was concluded that there were no overlapping roles between SakA and MpkC pathways.

We studied the phenotypic responses of *A. fumigatus *wild type and MAPK mutants, *sakA*Δ and *mpkC*Δ, to the seven benzaldehydes. We wanted to determine if these compounds, like in *S. cerevisiae*, targeted the cellular antioxidation system in a filamentous fungus. Both mutants were more sensitive, showing no growth, when treated with 0.8 mM 3,5-dimethoxybenzaldehyde (Figure 2D). The wild type strain maintained some growth, but with a 66% reduction in radial growth compared to the control. Similarly, when treated with 2,5- or 2,3-dimethoxybenzaldehyde at 0.6 mM or 2.0 mM, respectively, the *sakA*Δ and *mpkC*Δ mutants showed no growth, whereas the wild type showed only a 42% or 58% reduced growth, respectively (Figure data not shown). Other benzaldehydes also inhibited the growth of the *A. fumigatus *MAPK mutants. Amount of compound and Vincent equation % growth inhibition compared to the wild type (AF293: *sakA*Δ: *mpkC*Δ) are as follows: cinnamaldehyde at 0.4 mM (42: 59: 76), *o*-vanillin at 0.3 mM (40: 88: 58), 2-hydroxy-5-methoxybenzaldehyde at 0.4 mM (58: 65: 71), and 2-methoxybenzaldehyde at 0.8 mM (14: 16: 20) (Figure data not shown).

Thus, similar to that of the antioxidation mutants of *S. cerevisiae *(Figure 2B), the *sakA*Δ and *mpkC*Δ mutants of *A. fumigatus *were more sensitive to the benzaldehydes than the wild type. These results also indicated SakA and MpkC MAPK pathways may have overlapping roles in response to the antifungal activity of benzaldehyde analogs.

Dimethyldithiocarbamate (DDC) is a Cu,Zn-SOD inhibitor [54]. Since an equivalent mutant to *S. cerevisiae sod1*Δ is not currently available in *A. fumigatus*, we reasoned that treating *A. fumigatus *with DDC would chemically induce a phenotypic mimic of the *S. cerevisiae sod1*Δ mutant. In a separate group of experiments, we co-applied DDC with each of the seven benzaldehydes (Groups A - C) against *A. fumigatus *AF293. Co-application of the benzaldehyde derivatives with DDC increased inhibition of fungal growth of the compounds alone, in all combinations. These increases were from a low of 2% (DDC+ 2,3-dimethoxybenzaldehyde) to a high of 70% (DDC + 3,5-dimethoxybenzaldehyde), compared to the level of growth inhibition from the compounds, alone (Additional file 1 : TableS1). These increases of growth inhibition under the co-application of the compounds with an SOD inhibitor (DDC) were similar to what we observed in the yeast dilution bioassays of the compounds against the *S. cerevisiae sod1*Δ mutant (Figure 2B). There is one exception. While co-application of 2-methoxybenzaldehyde and DDC resulted in a 42% increase of growth inhibition in *A. fumigatus *(Additional file 1 : TableS1), the *sod1*Δ mutant was discernibly insensitive to this compound (Figure 2B). This insensitivity of the *sod1*Δ strain to 2-methoxybenzaldehyde may reflect the weaker antifungal activity of this compound compared to the other benzaldehydes identified.

The results with the *S. cerevisiae *mutants and with treatment of the *A. fumigatus *wild type with DDC indicate that both yeast and filamentous fungi respond similarly. In both types of fungi the cellular antioxidation system is a molecular target of the identified benzaldehyde analogs. The verification of the response of both a yeast and filamentous fungus to the benzaldehydes indicated the antioxidation system is a promising target to debilitate in order to increase effectiveness of fungal control agents.

### Chemosensitization of phenylpyrrole fungicides: using benzaldehydes to overcome fludioxonil tolerance of *A. fumigatus *MAPK mutants

**Figure 3:**
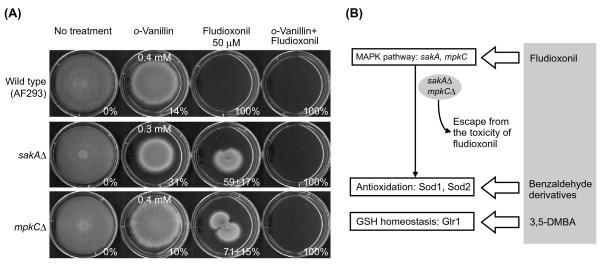
**Overcoming fludioxonil tolerance of *A. fumigatus *MAPK mutants (*sakA*Δ and *mpkC*Δ) by chemosensitization**. **(A) **Chemosensitization by using *o*-vanillin (%: Growth inhibition rate, SD < 5% except where noted). **(B) **Diagram showing the strategy for efficient control of fungal pathogens by using screened benzaldehyde analogs. 3,5-DMBA, 3,5-Dimethoxybenzaldehyde.

Certain fungi with mutations in genes involved in signal transduction of stress response, MAPK signaling pathway, can escape toxicity of the commercial fungicide fludioxonil [55]. Fludioxonil is a phenylpyrrole compound having a mode of action in fungi that triggers excessive stimulation of the normal, intact MAPK signaling pathway for glycerol biosynthesis [55]. The over-production of glycerol results in an "energy drain" that eventually inhibits fungal growth. We found *sakA*Δ and *mpkC*Δ, MAPK mutants of *A. fumigatus*, to be tolerant to 50 μM fludioxonil, resulting in only approximately 60% growth inhibition (Figure 3A). However, co-application of sub-fungicidal levels of *o*-vanillin with fludioxonil resulted in effective chemosensitization. The *o*-vanillin plus fludioxonil pairing did not allow these mutants to develop tolerance to fludioxonil, resulting in 100% mortality (Figure 3A).

Other benzaldehydes from Groups A - C were also tested for chemosensitization capacity in combination with fludioxonil (Data not shown). These combinations also resulted in loss of tolerance of these strains to fludioxonil. It is likely that benzaldehydes directly target genes in the antioxidation system, such as Cu,Zn-SOD, Mn-SOD, glutathione homeostasis, *etc*. These genes are downstream of their respective MAPK signaling pathways [53,56]. Hence, the chemosensitization by the benzo analogs results from bypassing the MAPK mutations and directly stressing the fungal antioxidative system (*e.g*., enzymes, *etc*.). The MAPK mutations, which had allowed tolerance to fludioxonil, now, are unable to respond to additional oxidative stress. This then results in inhibition of fungal growth by redox-active benzaldehydes (Figure 3B).

### Chemosensitization of inhibitors of complex II or III in MRC by using benzaldehyde analogs

Next, we tested chemosensitizing activity of three benzaldehyde analogs (the Group A compounds showing the highest antifungal activity) in co-applications with inhibitors, carboxin or AntA, of complex II or III, respectively, the best targets in MRC (Figure 1B). Co-treatment of cinnamaldehyde, *o*-vanillin or 2-hydroxy-5-methoxybenzaldehyde with AntA (a complex III inhibitor) mainly produced additive or synergistic interactions, depending on the fungal strains (FICIs, Table 3). The exceptions were *A. flavus *(cinnamaldehyde + AntA) or *A. fumigatus *(all treatments), which yielded neutral interactions.

**Table 3:** Antifungal interactions (FICI) of benzaldehyde derivatives tested alone or in combination with antimycin A or carboxin in microtiter plates^1^.

Compounds	MIC alone	MIC combined	FICI	MIC: alone	MIC: combined	FICI	MIC: alone	MIC: combined	FICI
**Strains**	***A. terreus *UAB698**			***A. terreus *UAB680**			***A. terreus *UAB673**		
Cinnamaldehyde Antimycin A	0.2 - 0.4 > 128 ^2^	0.1 - 0.2 8 - 16	0.56 A	0.2 - 0.4 > 128	0.1 - 0.2 4 - 8	0.53 A	0.2 - 0.4 > 128	0.1 - 0.2 4 - 8	0.53 A
*o*-Vanillin Antimycin A	0.2 - 0.4 > 128	0.1 - 0.2 4 - 8	0.53 A	0.2 - 0.4 > 128	0.1 - 0.2 4 - 8	0.53 A	0.2 - 0.4 > 128	0.1 - 0.2 4 - 8	0.53 A
2-Hydroxy-5-methoxy Antimycin A	0.2 - 0.4 > 128	0.1 - 0.2 64 - 128	1.00 A	0.2 - 0.4 > 128	0.1 - 0.2 8 - 16	0.56 A	0.2 - 0.4 > 128	0.1 - 0.2 8 - 16	0.56 A

**Strains**	***A. flavus *NRRL3357**			***A. fumigatus *AF293**			***P. expansum *NRRL974**		
Cinnamaldehyde Antimycin A	0.2 - 0.4 > 128	0.2 - 0.4 > 128	2.00 N	0.2 - 0.4 > 128	0.2 - 0.4 > 128	2.00 N	0.2 - 0.4 > 128	0.05 - 0.1 8 - 16	0.31 S
*o*-Vanillin Antimycin A	0.2 - 0.4 > 128	0.1 - 0.2 16 - 32	0.63 A	0.2 - 0.4 > 128	0.2 - 0.4 > 128	2.00 N	0.2 - 0.4 > 128	0.05 - 0.1 4 - 8	0.28 S
2-Hydroxy-5-methoxy Antimycin A	0.2 - 0.4 > 128	0.1 - 0.2 64 - 128	1.00 A	0.2 - 0.4 > 128	0.2 - 0.4 > 128	2.00 N	0.2 - 0.4 > 128	0.05 - 0.1 4 - 8	0.28 S
									

**Strains**	***A. terreus *UAB698**			***A. terreus *UAB680**			***A. terreus *UAB673**		
Cinnamaldehyde Carboxin	0.2 - 0.4 > 128 ^2^	0.2 - 0.4 > 128	2.00 N	0.2 - 0.4 > 128	0.2 - 0.4 > 128	2.00 N	0.2 - 0.4 > 128	0.2 - 0.4 > 128	2.00 N
*o*-Vanillin Carboxin	0.2 - 0.4 > 128	0.1 - 0.2 64 - 128	1.00 A	0.1 - 0.2 > 128	0.1 - 0.2 > 128	2.00 N	0.2 - 0.4 > 128	0.1 - 0.2 64 - 128	1.00 A
2-Hydroxy-5-methoxy Carboxin	0.2 - 0.4 > 128	0.1 - 0.2 64 - 128	1.00 A	0.2 - 0.4 > 128	0.1 - 0.2 32 - 64	0.75 A	0.2 - 0.4 > 128	0.1 - 0.2 16 - 32	0.63 A

**Strains**	***A. flavus *NRRL3357**			***A. fumigatus *AF293**			***P. expansum *NRRL974**		
Cinnamaldehyde Carboxin	0.4 - 0.8 > 128	0.2 - 0.4 64 - 128	1.00 A	0.2 - 0.4 > 128	0.2 - 0.4 > 128	2.00 N	0.2 - 0.4 > 128	0.1 - 0.2 64 - 128	1.00 A
*o*-Vanillin Carboxin	0.4 - 0.8 > 128	0.2 - 0.4 64 - 128	1.00 A	0.2 - 0.4 > 128	0.2 - 0.4 > 128	2.00 N	0.4 - 0.8 > 128	0.2 - 0.4 16 - 32	0.63 A
2-Hydroxy-5-methoxy Carboxin	0.4 - 0.8 > 128	0.2 - 0.4 32 - 64	0.75 A	0.2 - 0.4 > 128	0.2 - 0.4 > 128	2.00 N	0.2 - 0.4 > 128	0.2 - 0.4 > 128	2.00 N

^1^Compound interactions were determined as Fractional Inhibitory Concentration Indices (FICI), described by Isenberg ([47]; See also Methods). For calculation purposes, the higher concentration in each column was used. A, additive; N, neutral; S, synergistic. Benzaldehyde derivatives (mM)- cinnamaldehyde, *o*-vanillin, 2-hydroxy-5-methoxybenzaldehyde; Complex II inhibitor (μg/ml)- Carboxin; Complex III inhibitor (μg/ml)- Antimycin A.

^2^Since antifungal test was performed up to 128 μg/ml of antimycin A or carboxin (See Methods), 256 μg/ml (doubling of 128 μg/ml) was used for calculation purposes.

Co-application of Group A compounds with carboxin (a complex II inhibitor) showed less antifungal efficacy than when co-applied with AntA (Table 3). All interactions were additive in *A. flavus*. However, in the *A. terreus *strains and *P. expansum *only additive or neutral interactions occurred. As observed with the AntA co-applications, all interactions of the Group A benzo analogs with carboxin tested in *A. fumigatus *were neutral. These results indicate that MRC is probably a poorer target for chemosensitization in *A. fumigatus *than in the other fungi.

Collectively, our results with the MRC inhibitors and co-applied benzaldehydes show that the level of antifungal interaction depends on species and strain of fungus tested. However, overall, co-application of conventional MRC inhibitors with certain natural benzaldehydes did result in some promising interactions, *i.e*., synergistic/additive (Table 3).

### Benzaldehyde analogs as chemosensitizing agents to 2,3-dihydroxybenzaldehyde or thymol, inhibitors of cell wall/membrane integrity

In a prior study, two other natural phenolics, 2,3-dihydroxybenzaldehyde (2,3-D) and thymol, were found to interfere with fungal cell wall/membrane integrity [46]. Based on this mode of action, we reasoned that the newly identified benzaldehydes should be able to access target sites in fungi more effectively when combined with either 2,3-D or thymol. The chemosensitizing activities of the seven benzaldehydes were, thus, examined in combination with 2,3-D and thymol in all six filamentous fungal strains. The hypothesis was that these combinations should result in higher, perhaps even synergistic, antifungal activity.

Combinations of 2,3-D and the Group A - C benzaldehydes resulted in nine synergistic interactions with all others being additive, depending on the compound and strain (Additional file 2: TableS2). Hence, all of these co-applications resulted in increased antifungal activities relative to the individual application of each compound, alone, with no antagonistic or neutral interactions. Combinations of 2,3-D with *o*-vanillin, 2-hydroxy-5-methoxy-, 2,3-dimethoxy-, 2,5-dimethoxy- or 2-methoxy-benzaldehyde resulted in at least one synergistic interaction. Some synergistic interactions occurred with most of the *Aspergillus *species/strains. All interactions with *P. expansum *or *A. terreus *UAB698 were additive.

Combinations of thymol with the screened benzaldehyde analogs all resulted in additive interactions, with the exception of cinnamaldehyde in *A. flavus *(synergistic) (Additional file 3: TableS3). As with 2,3-D, our results indicate that the identified benzaldehydes with thymol increased antifungal activity of each compound when combined, with no antagonistic or neutral interactions.

### Formulation studies: inhibition of fungal growth by using mixtures of benzo analogs

Finally, we performed a formulation study of antifungal activity against the filamentous fungi, in which only natural benzo analogs were used. The purpose of this phase of our investigation was to determine if mixtures of our already identified benzo analogs could yield an effective level of antifungal activity, *in vitro*, against fungal pathogens, without a conventional antifungal drug. Formulations included the cell wall/membrane inhibitory compounds, 2,3-D and thymol, with the three Group A benzaldehydes (*o*-vanillin, 2-hydroxy-5-methoxybenzaldehyde and cinnamaldehyde).

Antifungal activities (*i.e*., MICs, MFCs, FE_MIC_s, FE_MFC_s; See Methods) of each of the benzo analogs, individually and combined as a formulation, against each of the strain/species of filamentous fungus tested are summarized in Table 4. In all cases, when each compound was applied individually, higher concentrations (*viz*., higher MICs) were required for the complete inhibition of fungal growth compared to the combination of all compounds in formulations. In some cases complete inhibition of fungal growth was not achieved at the highest concentration tested (128 μg/ml) by the individual compounds. MICs (microtiter) for the individual compounds were as follows: 64 μg/ml (*o*-vanillin, 2-hydroxy-5-methoxybenzaldehyde, cinnamaldehyde) for all strains; > 128 μg/ml (thymol) for all strains; 16 μg/ml (2,3-D) for *A. fumigatus *AF293/*A. terreus *UAB673; 32 μg/ml (2,3-D) for *A. terreus *UAB680; 64 μg/ml (2,3-D) for *A. terreus *UAB698/*P. expansum *NRRL974; 128 μg/ml (2,3-D) for *A. flavus *NRRL3357 (Table 4). However, formulations, which included mixtures of all five compounds, completely inhibited the growth of *A. fumigatus *AF293 or *A. terreus *UAB673 at an MIC of 4-8 μg/ml of each compound combined. Slightly higher MICs (8-16 μg/ml of each compound) were needed to achieve complete growth inhibition of *A. terreus *UAB680/UAB698, *A. flavus *NRRL3357 or *P. expansum *NRRL974 (Table 4).

**Table 4:** Fungicidal efficacy of benzaldehyde formulations (μg/ml) against individual strains of filamentous fungi examined^1^.

		Compounds alone						
				
		Cinnamaldehyde	*o*-Vanillin	2-Hydroxy-5-methoxybenzaldehyde	2,3-D	Thymol	Formulation (combined)	**Fungicidality MFC**_**FORMULATION**_**/MIC**_**FORMULATION**_
***A. fumigatus*** **AF293**	**MIC** (FE_MIC_) MFC (FE_MFC_)	32-64 (8 H) 64-128 (4 H)	32-64 (8 H) > 128 (8 H)	32-64 (8 H) > 128 (8 H)	8-16 (2 L) > 128 (8 H)	> 128 ^2^ (32 H) N/D ^3^ (8 H)	4-8 16-32	4 (Fungicidal)^4^
***A. terreus*** **UAB673**	**MIC** (FE_MIC_) MFC (FE_MFC_)	32-64 (8 H) 64-128 (4 H)	32-64 (8 H) > 128 (8 H)	32-64 (8 H) > 128 (8 H)	8-16 (2 L) > 128 (8 H)	> 128 (32 H) N/D (8 H)	4-8 16-32	4 (Fungicidal)
***A. terreus*** **UAB680**	**MIC** (FE_MIC_) MFC (FE_MFC_)	32-64 (4 H) 64-128 (4 H)	32-64 (4 H) > 128 (8 H)	32-64 (4 H) > 128 (8 H)	16-32 (2 L) > 128 (8 H)	> 128 (16 H) N/D (8 H)	8-16 16-32	2 (Fungicidal)
***A. terreus*** **UAB698**	**MIC** (FE_MIC_) MFC (FE_MFC_)	32-64 (4 H) 64-128 (4 H)	32-64 (4 H) > 128 (8 H)	32-64 (4 H) > 128 (8 H)	32-64 (4 H) > 128 (8 H)	> 128 (16 H) N/D (8 H)	8-16 16-32	2 (Fungicidal)
***A. flavus*** **NRRL3357**	**MIC** (FE_MIC_) MFC (FE_MFC_)	32-64 (4 H) 64-128 (4 H)	32-64 (4 H) > 128 (8 H)	32-64 (4 H) > 128 (8 H)	64-128 (8 H) > 128 (8 H)	> 128 (16 H) N/D (8 H)	8-16 16-32	2 (Fungicidal)
***P. expansum*** **NRRL974**	**MIC** (FE_MIC_) MFC (FE_MFC_)	32-64 (4 H) 32-64 (2 L)	32-64 (4 H) > 128 (8 H)	32-64 (4 H) > 128 (8 H)	32-64 (4 H) > 128 (8 H)	> 128 (16 H) N/D (8 H)	8-16 16-32	2 (Fungicidal)

^1 ^Efficacy of combined compounds compared to each compound alone is expressed as Formulation Efficacy (FE_MIC _or FE_MFC_; See Methods). FE_MIC_, FE for MIC values; FE_MFC_, FE for MFC values; H (high) if FE ≥ 4, L (low) if 1 < FE < 4; See Methods for calculations. For calculation purposes, higher concentration (μg/ml) of each concentration range was used for FE determination (*e.g*., 64 from 32 - 64 μg/ml).

^2^Assays were conducted up to a highest concentration of 128 μg/ml. For calculation purposes, 256 μg/ml (doubling of 128 μg/ml) was used.

^3^ND: Not determined since fungal growth occurred at the highest concentration (128 μg/ml). For calculation purposes, 256 μg/ml was used.

^4^Fungicidal or fungistatic effect was determined based on MFC_FORMULATION_/MIC_FORMULATION _ratio (Fungicidal if ≤ 4, fungistatic if > 4; See Methods for calculations). For calculation purposes, higher concentration (μg/ml) of each MIC or MFC range was used.

On the other hand, complete fungal kill (MFC) was not truly achieved with almost any of the compounds, individually (all MFCs > 128 μg/ml). The only exception was for cinnamaldehyde where the MFC was 64-128 μg/ml for all fungi except for *P. expansum *where the MFC was 32-64 μg/ml. However, when combined in formulations, all fungi were completely killed at an MFC of 16-32 μg/ml. Based on ratios of MFCs *vs*. MICs of the formulations (*i.e*., MFC_FORMULATION_/MIC_FORMULATION _≤ 4; See Table 4), all formulations were classified as "fungicidal" against all strains. The MICs of the formulations were, in some cases, > 10 times lower than those of the compounds treated individually. Hence, combining these benzo analogs in a formulation achieved *in vitro *antifungal activity equivalent or within an order of magnitude (at a μg/ml level) to that of currently available antifungal drugs (*e.g*., [57]).

Lastly, the potential contribution to formulation efficacy (FE_MIC _or FE_MFC _reflecting individual compound antifungal activity *vs*. in a formulation; See Methods) of the individual compounds was calculated to be high (H) (FEs ≥ 4) for cinnamaldehyde, *o*-vanillin, 2-hydroxy-5-methoxybenzaldehyde, 2,3-D and thymol in almost all cases (Table 4). The only exceptions were the FE_MIC_s of 2,3-D for *A. fumigatus *AF293, *A. terreus *UAB673/UAB680 or FE_MFC_s of cinnamaldehyde for *P. expansum *NRRL974, where the values were low (L) (≤ 2).

## Discussion

Collectively, the results of this study show that certain benzaldehyde analogs can act as potent antifungal agents, or as chemosensitizing agents in concert with conventional antimycotic products, to augment their efficacy. Based on fungal gene deletion mutant bioassays, the benzaldehydes studied target the fungal cellular antioxidation system, including MAPK signaling or the antioxidation enzymes, Cu,Zn-SOD, Mn-SOD, or glutathione reductase. These benzaldehydes also enhance, as chemosensitizing agents, the *in vitro *activity of conventional antifungal chemicals, such as MRC inhibitors or phenylpyrrole agents, and also natural phenolics such as 2,3-D and thymol in filamentous fungi. Co-application of benzaldehydes with other antifungal drugs, or applied in formulations of only the benzaldehydes, resulted in complete inhibition of fungal growth at much lower doses than any of the individual components applied, alone. Use of these benzaldehydes as chemosensitizing agents overcomes fungal tolerance to conventional fungicides, such as fludioxonil, and lowers dosage levels of conventional antifungal agents required for effective control.

Many antimicrobial drugs can disrupt the cellular antioxidation system of fungi. In this regard, such drugs can be considered as oxidative stress agents. Examples include MRC inhibitors and GSH depleting agents. For instance, C9-UK-2A, a structural derivative of AntA, showed potent antifungal activity against fungal pathogens, including *S. cerevisiae *[21,58]; pathogenic strains of *S. cerevisiae *have been isolated [58]. This drug triggers membrane injury, and induces the generation of cellular ROS against *Rhodotorula mucilaginosa *cells. C9-UK-2A inhibited the vegetative growth of *S. cerevisiae*, which also accompanies cellular and mitochondrial ROS generation [21]. This generation of ROS was due to the inhibition of electron flow at complex III in MRC. Meanwhile, treatment of fungal cells with dimethyldithiocarbamic acid or thiram [bis(dimethylthiocarbamoyl) disulfide] resulted in a rapid decrease in the level of cellular GSH, an important cellular antioxidant [59]. Consequently, the decrease in GSH will result in oxidative stress to fungi.

Other types of fungal oxidative stress agents include the phenylpyrroles, such as fludioxonil. In fungi, detection of environmental stresses, such as osmotic or oxidative stress, or cell wall disruption, is integrated into MAPK signaling pathways, which regulate downstream genes that are responsible for countering the stress [60,61]. Noteworthy is that mutations in fungal MAPK pathways, or upstream two-component (His-Asp phosphorelays) signaling systems, which relay environmental cues to the MAPK system, can result in tolerance to antifungal agents [55,62,63]. For example, fludioxonil interferes with fungal signaling systems resulting in excessive stimulation of the intact histidine kinase (HK)-MAPK stress-response pathway or glycerol biosynthesis [55,64,65]. This stimulation is akin to the osmotic stress response, which is also linked to cellular oxidative stress ([66] and references therein). However, studies have shown that if there is a mutation in the HK-MAPK signaling system, a fungus becomes resistant to fludioxonil [55,64]. Hence, an intact MAPK system is required for these types of fungicides to be effective. The *sakA*Δ and *mpkC*Δ MAPK mutants of *A. fumigatus *we used in our study are vivid examples of how such mutants can be tolerant to phenylpyrroles. However, we were able to show that by applying an oxidative stress agent (such as one of the benzaldehydes), these tolerant strains became susceptible because their mutated MAPK system was incapable of launching a fully operational oxidative stress response.

Involvement of stress-inducible protein(s) in drug resistance has already been documented [67,68]. The heat shock protein Hsp90, an essential molecular chaperone and key regulator of cell signaling, regulates folding, transport, maturation, and degradation of cellular proteins [69-71]. In both *S. cerevisiae *and the opportunistic yeast pathogen *Candida albicans*, Hsp90 potentiates rapid evolution of drug resistance to azoles [68]. Alternatively, drug resistance was abrogated by applying inhibitors of Hsp90 [67]. Hsp90 production is induced under stress, but availability of this protein may dwindle as there is increased cellular demand as the stress continues [72].

The scenario with Hsp90 is conceptually analogous to our findings presented here. As mentioned earlier, the fungal antioxidation enzymes, such as Cu,Zn-SOD, Mn-SOD, glutathione reductase, *etc*., are necessary to contend with adverse conditions generated by oxidative stress drugs (*e.g*., MRC inhibitors). Accordingly, cellular demand for these enzymes can continuously increase as more and more oxidative stress is applied. Hence, redox-active benzaldehydes, such as those in our study, can be useful chemosensitizing agents when co-applied with oxidative stress drugs. In this case the fungal antioxidation system would be overwhelmed because levels of antioxidation enzymes would not be sufficient for detoxification of the concerted activities of multiple oxidative stress agents (*e.g*., oxidative stress drug + redox-active chemosensitizers). Future studies may warrant a phenotypic-response screening of the entire set of deletion mutants of *S. cerevisiae *to the benzaldehydes. There is a possibility of other cellular targets to these compounds, in addition to the antioxidation system identified in our study.

Use of antifungal agents that are MRC inhibitors can also be toxic to mammalian cells. For example, TTFA (a complex II inhibitor) can directly decrease cellular respiration, and disrupt mitochondrial membrane potential in mammalian cells [73]. Also, TTFA delays cell cycle progression, which leads to an increase of cellular ROS, glutathione oxidation and a decrease in cellular ATP levels [73]. Similarly, AntA (a complex III inhibitor) inhibits mitochondrial respiration in rat liver cells, and increases production of cellular ROS ([74] and references therein). However, potential side effects of MRC inhibitors as antifungal agents can be reduced if effective dosage levels of MRC inhibitors can be diminished. Such a reduction can be achieved by use of redox-active natural phenolics as chemosensitizers to MRC inhibitors, as shown in our study.

MRC inhibitors can also be used to enhance antifungal drugs. For example, co-application of MRC inhibitors with the antifungal drug, caspofungin (an inhibitor of cell wall integrity), greatly increased susceptibility of *Candida parapsilosis *to caspofungin [75]. Contrastingly, inducing the alternative respiratory pathway in MRC results in decreasing the susceptibility of *C. albicans *to antifungal triazole drugs [76]. Thus, use of MRC inhibitors (along with chemosensitizing agents) should be considered further for effective control of human mycoses. Another form of fungal defense, involving multidrug resistance, can also be disrupted to enhance antifungal activity. For example, in strains of *S. cerevisiae *where multidrug resistant genes *PDR1*, *PDR3*, or *PDR5 *are mutated, there is increased sensitivity to mucidin, an MRC inhibitor [20].

## Conclusions

Cellular antioxidation systems appear to be promising molecular targets of natural phenolics for the effective control of fungi. Benzaldehyde analogs, such as those identified in this study, can be used as potent chemosensitizing agents to enhance anitmycotic activity of already available antifungal drugs. Our study focused on the effects of the tested benzaldehydes against the selected filamentous fungi. They also showed similar effects on the strains of *S. cerevisiae*, used in our study to examine mode of action. However, it is likely these benzaldehydes would have similar activity against pathogenic yeasts, such as *Candida *species and *Cryptococcus neoformans*. Certain benzaldehydes were recently reported to have chemosensitizing activity, in combination with certain antifungal drugs (amphotericin B, triazoles), against reference strains of *C. albicans *and *C. neoformans *[77]. Correspondingly, the benzaldehydes examined in our study, here, have potent antifungal activity against clinical strains of these yeasts (manuscript in preparation). Such chemosensitization can reduce costs, lower resistance, and alleviate health risks associated with current antifungal therapy. Further *in vivo *studies are necessary to determine if the *in vitro *activities demonstrated herein can translate to clinically effective and safe chemotherapeutic resolution of mycoses.

## Abbreviations

AntA: Antimycin A; AOX: Alternative oxidase; BHAM: Benzhydroxamic acid; CLSI: Clinical Laboratory Standards Institute; Cu,Zn-SOD: Cytosolic superoxide dismutase; 2,3-D: 2,3-Dihydroxybenzaldehyde; DDC: Dimethyldithiocarbamate; DMSO: Dimethylsulfoxide; FFCI: Fractional Fungicidal Concentration Indices; FICI: Fractional Inhibitory Concentration Indices; GSH: Glutathione (reduced form); GSSG: Glutathione (oxidized form); KCN: Potassium cyanide; Kre-Me: Kresoxim methyl; MAPK: Mitogen-Activated Protein Kinase; MIC: Minimum Inhibitory Concentration; MFC: Minimum Fungicidal Concentration; Mn-SOD: Mitochondrial superoxide dismutase; MRC: Mitochondrial respiratory chain; Na-azide: Sodium azide; 3-NPA: 3-Nitropropionic acid; PCS: Pyraclostrobin; PDA: Potato dextrose agar; ROS: Reactive oxygen species; SG medium: Synthetic glucose medium; Rot: Rotenone; SHAM: Salicylhydroxamic acid; TTFA: Thenoyltrifluoroacetone.

## Competing interests

The authors declare that they have no competing interests.

## Authors' contributions

JHK designed and performed research including data analysis and interpretation, literature search, and wrote the manuscript. KLC and NM performed antifungal assays and prepared the figures and the final manuscript. BCC directed research and revised the manuscript. All authors have read and approved the final manuscript.

## Supplementary Material

Additional file 1 **Table S1. Enhanced growth inhibition of *Aspergillus fumigatus *AF293 by co-application of benzaldehyde derivatives and diethyldithiocarbamate (DDC)^1^**. ^1 ^Number in each column indicates % inhibition of fungal radial growth, which was based on Vincent equation (See Methods) (SD < 5%). Diethyldithiocarbamate (DDC): Cu,Zn-SOD (Cytosolic superoxide dismutase) inhibitor. ^2 ^The value of % increase by co-application (*i.e*., compound + DDC) = (Vincent eq. value from co-application) - (Vincent eq. value from independent treatment, *i.e*., compound or DDC alone, showing higher % growth inhibition). Click here for file

Additional file 2 **Table S2. Antifungal interactions (FICI) of 2,3-dihydroxybenzaldehyde (2,3-D; mM) and other benzaldehyde derivatives (mM) tested alone or in combination in microtiter plates^1^**. ^1 ^Compound interactions were determined as Fractional Inhibitory Concentration Indices (FICI), described by Isenberg ([47]; See Methods). For calculation purposes, the higher concentration in each column was used. A, additive; N, neutral; S, synergistic. Click here for file

Additional file 3 **Table S3. Antifungal interactions (FICI) of thymol (mM) and benzaldehyde derivatives (mM) tested alone or in combination in microtiter plates^1^**. ^1 ^Compound interactions were determined as Fractional Inhibitory Concentration Indices (FICI), described by Isenberg ([47]; See Methods). For calculation purposes, the higher concentration in each column was used. A, additive; N, neutral; S, synergistic. Click here for file
