# Supplementary material for: Antifungal activity of redox-active benzaldehydes that target cellular antioxidation
Source: Ann Clin Microbiol Antimicrob. 2011 May 31;10:23. doi: 10.1186/1476-0711-10-23 (PMC3127747; doi:10.1186/1476-0711-10-23)
Supplement: Additional file 1 — Table S1. Enhanced growth inhibition of Aspergillus fumigatus AF293 by co-application of benzaldehyde derivatives and diethyldithiocarbamate (DDC)1. 1 Number in each column indicates % inhibition of fungal radial growth, which was based on Vincent equation (See Methods) (SD < 5%). Diethyldithiocarbamate (DDC): Cu,Zn-SOD (Cytosolic superoxide dismutase) inhibitor. 2 The value of % increase by co-application (i.e., compound + DDC) = (Vincent eq. value from co-application) - (Vincent eq. value from independent treatment, i.e., compound or DDC alone, showing higher % growth inhibition). [file 1476-0711-10-23-S1.PDF]

TableS1. Enhanced growth inhibition of *Aspergillus fumigatus* AF293 by co-application of benzaldehyde derivatives and diethyldithiocarbamate (DDC).[1]

Compound	No treat	Compd alone	DDC (0.5 mM) alone	Combined (Compd + DDC)	% Increase in growth inhibition by co-application[2]
**Cinnamaldehyde** (0.3 mM)	0	18	10	30	12
***o*-Vanillin** (0.3 mM)	0	40	2	60	20
**2-OH-5-methoxy-benzaldehyde** (0.3 mM)	0	20	6	72	52
**2,5-Dimethoxy-benzaldehyde** (0.4 mM)	0	16	10	46	30
**3,5-Dimethoxy-benzaldehyde** (0.6 mM)	0	10	2	80	70
**2,3-Dimethoxy-benzaldehyde** (1.2 mM)	0	12	2	14	2
**2-Methoxy-benzaldehyde** (0.8 mM)	0	14	14	56	42

[1] Number in each column indicates % inhibition of fungal radial growth, which was based on Vincent equation (See Methods) (SD < 5%). Diethyldithiocarbamate (DDC): Cu,Zn-SOD (Cytosolic superoxide dismutase) inhibitor.

[2] The value of % increase by co-application (*i.e.*, compound + DDC) = (Vincent eq. value from co-application) - (Vincent eq. value from independent treatment, *i.e.*, compound or DDC alone, showing higher % growth inhibition).
