# Supplementary material for: Antifungal activity of redox-active benzaldehydes that target cellular antioxidation
Source: Ann Clin Microbiol Antimicrob. 2011 May 31;10:23. doi: 10.1186/1476-0711-10-23 (PMC3127747; doi:10.1186/1476-0711-10-23)
Supplement: Additional file 2 — Table S2. Antifungal interactions (FICI) of 2,3-dihydroxybenzaldehyde (2,3-D; mM) and other benzaldehyde derivatives (mM) tested alone or in combination in microtiter plates1. 1 Compound interactions were determined as Fractional Inhibitory Concentration Indices (FICI), described by Isenberg ([47]; See Methods). For calculation purposes, the higher concentration in each column was used. A, additive; N, neutral; S, synergistic. [file 1476-0711-10-23-S2.PDF]

TableS2. Antifungal interactions (FICI) of 2,3-dihydroxybenzaldehyde (2,3-D; mM) and other benzaldehyde derivatives (mM) tested alone or in combination in microtiter plates.[1]

Compounds	MIC alone	MIC combined	FICI	MIC: alone	MIC: combined	FICI	MIC: alone	MIC: combined	FICI
	A. terreus **UAB698**			***A. terreus*** **UAB680**			***A. terreus*** **UAB673**		
Cinnamaldehyde	0.4 - 0.8	0.2 - 0.4	0.75 A	0.2 - 0.4	0.1 - 0.2	1.00 A	0.2 - 0.4	0.0 - 0.1	0.75 A
2,3-D	0.2 - 0.4	0.0 - 0.1		0.1 - 0.2	0.0 - 0.1		0.1 - 0.2	0.0 - 0.1	
o-Vanillin	0.2 - 0.4	0.1 - 0.2	1.00 A	0.2 - 0.4	0.0 - 0.1	0.50 S	0.2 - 0.4	0.0 - 0.1	0.50 S
2,3-D	0.2 - 0.4	0.1 - 0.2		0.2 - 0.4	0.0 - 0.1		0.2 - 0.4	0.0 - 0.1	
2-Hydroxy-5-methoxy	0.2 - 0.4	0.1 - 0.2	1.00 A	0.2 - 0.4	0.05 - 0.1	0.50 S	0.2 - 0.4	0.1 - 0.2	0.75 A
2,3-D	0.2 - 0.4	0.1 - 0.2		0.2 - 0.4	0.05 - 0.1		0.1 - 0.2	0.0 - 0.05	
2,5-Dimethoxy	0.8 - 1.6	0.2 - 0.4	0.75 A	0.4 - 0.8	0.2 - 0.4	0.75 A	0.4 - 0.8	0.2 - 0.4	0.75 A
2,3-D	0.2 - 0.4	0.1 - 0.2		0.1 - 0.2	0.0 - 0.05		0.1 - 0.2	0.0 - 0.05	
3,5-Dimethoxy	0.8 - 1.6	0.2 - 0.4	0.75 A	0.4 - 0.8	0.2 - 0.4	0.75 A	0.2 - 0.4	0.1 - 0.2	0.75 A
2,3-D	0.2 - 0.4	0.1 - 0.2		0.1 - 0.2	0.0 - 0.05		0.1 - 0.2	0.0 - 0.05	
2,3-Dimethoxy	0.8 - 1.6	0.2 - 0.4	0.75 A	0.8 - 1.6	0.4 - 0.8	0.75 A	0.8 - 1.6	0.2 - 0.4	0.50 S
2,3-D	0.2 - 0.4	0.1 - 0.2		0.1 - 0.2	0.0 - 0.05		0.1 - 0.2	0.0 - 0.05	
2-Methoxy	1.6 - 3.2	0.2 - 0.4	0.63 A	0.8 - 1.6	0.2 - 0.4	0.50 S	0.8 - 1.6	0.1 - 0.2	0.38 S
2,3-D	0.2 - 0.4	0.1 - 0.2		0.1 - 0.2	0.0 - 0.05		0.1 - 0.2	0.0 - 0.05	
	A. flavus **NRRL3357**			***A. fumigatus*** **AF293**			***P. expansum*** **NRRL974**		
Cinnamaldehyde	0.2 - 0.4	0.1 - 0.2	1.00 A	0.4 - 0.8	0.2 - 0.4	1.00 A	0.2 - 0.4	0.1 - 0.2	1.00 A
2,3-D	0.2 - 0.4	0.1 - 0.2		0.05 - 0.1	0.0 - 0.05		0.2 - 0.4	0.1 - 0.2	
o-Vanillin	0.2 - 0.4	0.0 - 0.1	0.75 A	0.2 - 0.4	0.0 - 0.1	0.50 S	0.4 - 0.8	0.1 - 0.2	0.75 A
2,3-D	0.4 - 0.8	0.2 - 0.4		0.2 - 0.4	0.0 - 0.1		0.2 - 0.4	0.1 - 0.2	
2-Hydroxy-5-methoxy	0.2 - 0.4	0.1 - 0.2	1.00 A	0.2 - 0.4	0.1 - 0.2	0.75 A	0.2 - 0.4	0.1 - 0.2	0.75 A
2,3-D	0.2 - 0.4	0.1 - 0.2		0.1 - 0.2	0.0 - 0.05		0.2 - 0.4	0.05 - 0.1	
2,5-Dimethoxy	0.8 - 1.6	0.2 - 0.4	0.50 S	0.4 - 0.8	0.2 - 0.4	0.75 A	0.8 - 1.6	0.4 - 0.8	0.75 A
2,3-D	0.4 - 0.8	0.1 - 0.2		0.1 - 0.2	0.0 - 0.05		0.2 - 0.4	0.05 - 0.1	
3,5-Dimethoxy	0.8 - 1.6	0.4 - 0.8	0.63 A	0.4 - 0.8	0.2 - 0.4	1.00 A	0.8 - 1.6	0.2 - 0.4	0.75 A
2,3-D	0.4 - 0.8	0.05 - 0.1		0.05 - 0.1	0.0 - 0.05		0.2 - 0.4	0.1 - 0.2	
2,3-Dimethoxy	0.8 - 1.6	0.4 - 0.8	0.63 A	1.6 - 3.2	0.4 - 0.8	0.50 S	0.8 - 1.6	0.2 - 0.4	0.75 A
2,3-D	0.2 - 0.4	0.0 - 0.05		0.1 - 0.2	0.0 - 0.05		0.2 - 0.4	0.1 - 0.2	
2-Methoxy	0.8 - 1.6	0.4 - 0.8	0.63 A	0.8 - 1.6	0.4 - 0.8	0.75 A	0.8 - 1.6	0.4 - 0.8	0.75 A
2,3-D	0.2 - 0.4	0.0 - 0.05		0.1 - 0.2	0.0 - 0.05		0.2 - 0.4	0.05 - 0.1	

[1] Compound interactions were determined as Fractional Inhibitory Concentration Indices (FICI), described by Isenberg ([47]; See Methods). For calculation purposes, the higher concentration in each column was used. A, additive; N, neutral; S, synergistic.
