# Supplementary material for: Antifungal activity of redox-active benzaldehydes that target cellular antioxidation
Source: Ann Clin Microbiol Antimicrob. 2011 May 31;10:23. doi: 10.1186/1476-0711-10-23 (PMC3127747; doi:10.1186/1476-0711-10-23)
Supplement: Additional file 3 — Table S3. Antifungal interactions (FICI) of thymol (mM) and benzaldehyde derivatives (mM) tested alone or in combination in microtiter plates1. 1 Compound interactions were determined as Fractional Inhibitory Concentration Indices (FICI), described by Isenberg ([47]; See Methods). For calculation purposes, the higher concentration in each column was used. A, additive; N, neutral; S, synergistic. [file 1476-0711-10-23-S3.PDF]

TableS3. Antifungal interactions (FICI) of thymol (mM) and benzaldehyde derivatives (mM) tested alone or in combination in microtiter plates.[1]

**Compounds**	**MIC alone**	**MIC combined**	**FICI**	**MIC: alone**	**MIC: combined**	**FICI**	**MIC: alone**	**MIC: combined**	**FICI**
	***A. terreus* UAB698**			***A. terreus* UAB680**			***A. terreus* UAB673**		
Cinnamaldehyde	0.4 - 0.8	0.2 - 0.4	0.75 A	0.2 - 0.4	0.1 - 0.2	0.75 A	0.2 - 0.4	0.1 - 0.2	0.75 A
Thymol	0.8 - 1.6	0.2 - 0.4		0.8 - 1.6	0.2 - 0.4		0.8 - 1.6	0.2 - 0.4	
o-Vanillin	0.2 - 0.4	0.1 - 0.2	0.75 A	0.2 - 0.4	0.1 - 0.2	0.63 A	0.2 - 0.4	0.1 - 0.2	0.63 A
Thymol	0.8 - 1.6	0.2 - 0.4		0.8 - 1.6	0.1 - 0.2		0.8 - 1.6	0.1 - 0.2	
2-Hydroxy-5-methoxy	0.2 - 0.4	0.1 - 0.2	0.75 A	0.2 - 0.4	0.1 - 0.2	0.75 A	0.2 - 0.4	0.1 - 0.2	0.63 A
Thymol	0.8 - 1.6	0.2 - 0.4		0.8 - 1.6	0.2 - 0.4		0.8 - 1.6	0.1 - 0.2	
2,5-Dimethoxy	0.8 - 1.6	0.0 - 0.1	0.56 A	0.4 - 0.8	0.1 - 0.2	0.75 A	0.4 - 0.8	0.1 - 0.2	0.75 A
Thymol	0.8 - 1.6	0.4 - 0.8		0.8 - 1.6	0.4 - 0.8		0.8 - 1.6	0.4 - 0.8	
3,5-Dimethoxy	0.8 - 1.6	0.4 - 0.8	0.75 A	0.4 - 0.8	0.2 - 0.4	1.00 A	0.4 - 0.8	0.2 - 0.4	1.00 A
Thymol	0.8 - 1.6	0.2 - 0.4		0.8 - 1.6	0.4 - 0.8		0.8 - 1.6	0.4 - 0.8	
2,3-Dimethoxy	0.8 - 1.6	0.0 - 0.1	0.56 A	0.8 - 1.6	0.2 - 0.4	0.75 A	0.8 - 1.6	0.4 - 0.8	1.00 A
Thymol	0.8 - 1.6	0.4 - 0.8		0.8 - 1.6	0.4 - 0.8		0.8 - 1.6	0.4 - 0.8	
2-Methoxy	1.6 - 3.2	0.8 - 1.6	0.75 A	0.8 - 1.6	0.4 - 0.8	1.00 A	0.8 - 1.6	0.4 - 0.8	1.00 A
Thymol	0.8 - 1.6	0.2 - 0.4		0.8 - 1.6	0.4 - 0.8		0.8 - 1.6	0.4 - 0.8	
	***A. flavus* NRRL3357**			***A. fumigatus* AF293**			***P. expansum* NRRL974**		
Cinnamaldehyde	0.4 - 0.8	0.1 - 0.2	0.50 S	0.4 - 0.8	0.2 - 0.4	0.63 A	0.4 - 0.8	0.2 - 0.4	0.63 A
Thymol	0.8 - 1.6	0.2 - 0.4		0.4 - 0.8	0.0 - 0.1		0.4 - 0.8	0.0 - 0.1	
o-Vanillin	0.2 - 0.4	0.1 - 0.2	0.75 A	0.2 - 0.4	0.1 - 0.2	0.63 A	0.1 - 0.2	0.0 - 0.1	0.75 A
Thymol	0.4 - 0.8	0.1 - 0.2		0.4 - 0.8	0.0 - 0.1		0.4 - 0.8	0.1 - 0.2	
2-Hydroxy-5-methoxy	0.2 - 0.4	0.1 - 0.2	1.00 A	0.2 - 0.4	0.0 - 0.1	0.75 A	0.2 - 0.4	0.1 - 0.2	1.00 A
Thymol	0.4 - 0.8	0.2 - 0.4		0.4 - 0.8	0.2 - 0.4		0.4 - 0.8	0.2 - 0.4	
2,5-Dimethoxy	0.8 - 1.6	0.4 - 0.8	1.00 A	0.4 - 0.8	0.0 - 0.1	0.63 A	0.8 - 1.6	0.0 - 0.1	0.56 A
Thymol	0.4 - 0.8	0.2 - 0.4		0.4 - 0.8	0.2 - 0.4		0.8 - 1.6	0.4 - 0.8	
3,5-Dimethoxy	0.8 - 1.6	0.4 - 0.8	1.00 A	0.4 - 0.8	0.0 - 0.1	0.63 A	0.8 - 1.6	0.4 - 0.8	1.00 A
Thymol	0.4 - 0.8	0.2 - 0.4		0.4 - 0.8	0.2 - 0.4		0.4 - 0.8	0.2 - 0.4	
2,3-Dimethoxy	1.6 - 3.2	0.8 - 1.6	0.75 A	1.6 - 3.2	0.8 - 1.6	0.75 A	1.6 - 3.2	0.8 - 1.6	0.75 A
Thymol	0.4 - 0.8	0.1 - 0.2		0.4 - 0.8	0.1 - 0.2		0.8 - 1.6	0.2 - 0.4	
2-Methoxy	1.6 - 3.2	0.8 - 1.6	0.75 A	0.4 - 0.8	0.1 - 0.2	0.75 A	1.6 - 3.2	0.8 - 1.6	0.56 A
Thymol	0.4 - 0.8	0.1 - 0.2		0.4 - 0.8	0.2 - 0.4		0.8 - 1.6	0.0 - 0.1	

[1]Compound interactions were determined as Fractional Inhibitory Concentration Indices (FICI), described by Isenberg ([47]; See Methods). For calculation purposes, the higher concentration in each column was used. A, additive; N, neutral; S, synergistic.
